# Effects of Knee-Powered Exoskeleton-Assisted Gait Training on Walking Function and Symmetry in Subacute Stroke: A Randomized Pilot Trial

**DOI:** 10.3390/bioengineering13091059

**Published:** 2026-09-11

**Authors:** Yu-Ming Zhang, Ting-Yu Lin, Simon Fuk-Tan Tang, Hsiu-Chun Chen, Chun-Sheng Ho

**Affiliations:** 1 Department of Physical Medicine and Rehabilitation, Lo-Hsu Medical Foundation, Inc., Lotung Poh-Ai Hospital, Yilan 265501, Taiwant840326@icloud.com (T.-Y.L.); fttang1239@gmail.com (S.F.-T.T.);; 2 Graduate Institute of Clinical Medicine, College of Medicine, Taipei Medical University, Taipei 110301, Taiwan; 3 Department of Sports and Health Promotion, Fo Guang University, Yilan 26247, Taiwan

**Keywords:** stroke, exoskeleton device, gait analysis, rehabilitation

## Abstract

**Background:** Our objective was to evaluate the clinical effects on walking function and symmetry of combined knee-powered exoskeleton-assisted gait training (EAGT) and conventional physiotherapy versus conventional therapy alone in patients with subacute stroke. **Methods:** This randomized pilot trial enrolled patients 1–3 months post-stroke with Functional Ambulation Classification scores of 3 or 4. Participants were randomly assigned in a 1:1 ratio to receive EAGT plus conventional therapy or conventional therapy alone for 4 weeks. Outcomes included gait speed (10MWT), endurance (6MWT), and motor function (FMA-LE), assessed at baseline, 4 weeks, and 16 weeks. Accelerometer-based spatiotemporal gait parameters were analyzed in the EAGT group. **Results:** Twenty-four participants (EAGT: 17, control: 7) completed the study. No significant group × time interaction was observed for gait speed, walking endurance, or lower-extremity motor function, indicating that changes over time did not differ significantly between the EAGT and control groups. In the EAGT group, increased gait speed was accompanied by augmented cadence (*p* < 0.001) without significant changes in stride length or gait symmetry. **Conclusions:** From this preliminary pilot study, knee-powered EAGT improved walking performance in subacute stroke but did not demonstrate superiority over conventional rehabilitation. Gains were accompanied by cadence augmentation without restoration of gait symmetry. Larger randomized trials are needed to validate these preliminary findings.

## 1. Introduction

Stroke remains one of the leading causes of mortality and long-term disability worldwide. An estimated 30.7 million individuals are currently living with stroke, with prevalence rates ranging between 46.1 and 73.3 per 1000 population [1,2]. Each year, approximately 11.9 million new cases occur, resulting in more than 160.5 million disability-adjusted life-years lost [3]. Hemiplegia, impaired muscle strength, abnormal tone, and deficits in coordination are major contributors to post-stroke walking disability, which affects 80% of patients [4]. Restoring safe and efficient ambulation is a top priority for both patients and rehabilitation teams. Although the majority of patients eventually obtain some degree of independent walking, fewer than 10% achieve the capacity to ambulate outdoors, and fewer than 5% are able to ascend or descend stairs by the time of discharge from inpatient rehabilitation [5]. Even among those who regain basic mobility, persistent gait abnormalities—including reduced speed, cadence, and stride length as well as increased spatiotemporal asymmetry—are common [6,7]. Establishing a physiological gait pattern could improve energy efficiency and prevent musculoskeletal injuries caused by the excess stress placed on the non-paretic leg.

Functional recovery after stroke is mediated by cortical reorganization and the acquisition of compensatory strategies. Highly intensive and task-specific training is critical for driving neuroplasticity, particularly during the early post-stroke period [8]. Active participation is fundamental to elicit changes in motor performance and cortical activity [9]. Body weight-supported treadmill training has been developed to facilitate normal gait patterns [10]; however, this approach often necessitates the involvement of two or more physiotherapists. Recently, robot-assisted gait training has gained momentum, as it enables repetitive practice while reducing the physical strain on therapists. Meta-analyses have demonstrated that treadmill-based robotic systems yield improvements in gait velocity and lower-limb motor function comparable to conventional therapy [11,12]. Furthermore, a Cochrane review published in 2025 reported that combined electromechanical-assisted training and conventional physical therapy produce similar enhancements in walking velocity and functional capacity, and this combination increases the probability of achieving independent ambulation [13]. Stationary robotic walking systems employ a secure harness and pre-set body motion and gait speed, where voluntary contribution is limited [14]. Powered exoskeletons differ from stationary systems in that they require active engagement from the user. Equipped with sensors and algorithm-driven control mechanisms, these devices detect patient-generated movements and deliver motorized joint assistance in synchrony with the intended gait cycle. Patients must initiate the swing phase and control foot placement, while simultaneously maintaining an upright posture. Accordingly, selection of an optimal robotic design must be individualized based on the patient’s motor function level [10].

Among stroke patients who retain independent ambulation, strength of the paretic lower limb, particularly the knee extensors, has been associated with gait speed [15,16]. Studies have further shown that the peak torque and total work of the paretic knee extensors are correlated with both comfortable and fast walking speeds, with knee-extensor work being an important determinant of fast gait velocity [17]. Knee-extensor strength has also been associated with step-length asymmetry [17]. Nevertheless, post-stroke gait asymmetry is multifactorial and cannot be attributed to weakness at a single joint. Reduced paretic propulsion may result from impairments in ankle plantarflexion, knee extension, hip flexion, and paretic-limb extension during late stance and push-off, all of which can affect limb advancement, swing time, and step length [18]. Most wearable exoskeletons developed for post-stroke gait training have emphasized assistance at the hip or ankle, whereas the potential contribution of targeted knee assistance has received less attention. Because the knee extensors contribute to stance-phase limb support and progression, and their strength is associated with walking speed and step-length characteristics, assisting knee motion may provide a means of improving both walking capacity and gait symmetry.

“Keeogo” is a wearable, overground exoskeleton designed to help people with ambulation disabilities, whether it be due to neurological or orthopedic conditions [19,20,21,22]. This study aims to evaluate the clinical effects of combined knee-powered exoskeleton-assisted gait training (EAGT) and rehabilitation versus conventional rehabilitation alone for individuals with subacute stroke. Adaptations in temporal and spatial gait characteristics following EAGT will also be analyzed to elucidate the underlying changes in gait mechanisms induced by this intervention.

## 2. Materials and Methods

### 2.1. Study Design

A prospective, randomized clinical trial was conducted to compare the efficacy of EAGT plus conventional rehabilitation versus conventional rehabilitation alone in patients with subacute stroke.

### 2.2. IRB Approval Statement

This study was approved by the Research Ethics Committee of Hualien Tzu Chi Hospital, Buddhist Tzu Chi Medical Foundation Hospital (Approval Number: IRB110-192-A). The investigation was conducted in accordance with the ethical standards of the responsible committee on human experimentation and the Declaration of Helsinki. Written informed consent was obtained from all participants prior to their inclusion in the study.

### 2.3. Participants

Consecutive patients admitted for post-stroke rehabilitation were screened for eligibility. Inclusion criteria were: first-ever supratentorial stroke; onset 1 to 3 months before enrollment; age > 20 years; and a Functional Ambulation Classification (FAC) score of 3 or 4, indicating the ability to ambulate on level ground independently or under supervision. Exclusion criteria included: cerebellum or brainstem stroke; aphasia or cognitive impairment (Mini-Mental State Examination score < 24) precluding adherence to protocol; pre-stroke non-ambulatory status; osteoporosis; severe spasticity (Modified Ashworth Scale > 2 of any leg joint) or involuntary movements of the lower limbs, musculoskeletal or skin conditions preventing exoskeleton use (e.g., pain, contractures, fractures, and nonhealing pressure injuries); significant cardiopulmonary disease (resting blood pressure > 180/100 mm Hg, recent myocardial infarction, congestive heart failure, unstable arrhythmias, severe aortic stenosis, or angina/shortness of breath); severe orthostatic hypotension; and uncontrolled seizures. Written informed consent was obtained from all participants. Participants were randomly assigned in a 1:1 ratio to either the experimental or control group using a computer-generated random number table. Due to the inherent nature of the robotic intervention, double-blinding of participants and care providers was not feasible following allocation.

### 2.4. Exoskeleton Device

The lower extremity exoskeleton (Keeogo, B-Temia Inc., Wistron Medical Technology, New Taipei City, Taiwan) boosts mobility through biomechanical assistance and knee joint stabilization. The device facilitates flexion and extension of both knees during functional activities, delivering complementary torque in response to movement signals detected by sensors at the hip and knee joints. Motor activation occurs only after the user initiates a step. The level of assistance can be adjusted to match the patient’s own capabilities. The system braces the knee joint but does not impose a fixed gait pattern and gives no additional support to the trunk or ankles.

### 2.5. Intervention

All patients underwent 60 min of physical therapy per day, five days per week, over a four-week period. The experimental group received 30 min of EAGT combined with 30 min of conventional rehabilitation, whereas the control group received 60 min of conventional rehabilitation. For conventional rehabilitation, gait training was delivered by experienced physiotherapists and focused on improving gait quality and efficiency. Interventions included overground walking practice at different self-selected and faster speeds, dynamic postural control during walking, weight transfer and loading of the paretic limb, turning and changes in direction, and adaptation to varying walking demands. Verbal, visual, and manual cues were provided as needed to facilitate more symmetrical gait and appropriate lower-extremity alignment.

For the EAGT group, participants performed overground walking while wearing the exoskeleton, with emphasis on repetitive walking practice, active participation, appropriate weight transfer, knee control during stance and swing, and a more efficient and symmetrical gait pattern.

Training intensity was progressively increased by adjusting walking speed, titrating the level of robotic assistance, and extending the duration of continuous ambulation. Stair negotiation exercises were introduced for participants with higher functional capacity. In addition, all participants received 30 min of occupational therapy each training day.

### 2.6. Outcome Measures

Gait performance without wearing the exoskeleton was evaluated at three time points: baseline (prior to intervention), immediately post-training at 4 weeks, and follow-up at 12 weeks after the intervention ended. The assessment battery comprised: (1) gait speed, measured by the 10-m walk test (10MWT), which calculates comfortable walking speed (m/s) across a 10-m distance averaged over three trials, while excluding acceleration and deceleration phases; (2) walking endurance and functional exercise capacity, assessed via the 6-min walk test (6MWT), which records the total distance walked in meters along a flat 30-m corridor over 6 min at a self-paced speed; and (3) lower-limb motor impairment, evaluated using the Fugl–Meyer Assessment for the Lower Extremity (FMA-LE), which examines hip, knee, and ankle sensorimotor recovery across reflexive, synergic, and non-synergic movements using a 3-point ordinal scale (0 = unable to perform to 2 = complete performance; total score: 0–34) [23]. Additionally, for the EAGT group, spatiotemporal gait parameters were recorded using an accelerometer at each of the three evaluation intervals. These parameters included gait speed, cadence, and stride length. To ensure methodological consistency with established post-stroke gait assessment protocols, the symmetry index (SI) was employed to evaluate gait symmetry [24]. The SI was calculated for the stance phase, swing phase, and step length. For each spatiotemporal variable, the following equation was applied: SI = [(V_paretic_ − V_non-paretic_)/0.5(V_paretic_ + V_non-paretic_)] × 100%. All evaluations were conducted by a senior physiotherapist who was not involved in treating the study patients to ensure objective appraisal.

### 2.7. Data Analysis

Statistical analyses were conducted using SPSS version 27 (IBM Corp., Armonk, NY, USA). Continuous variables were expressed as mean ± standard deviation (SD) and compared using independent *t*-tests, while categorical variables were analyzed using Fisher’s exact test. To evaluate the intervention effects, a two-way mixed analysis of variance (ANOVA) was conducted with group (EAGT vs. control) as the between-subjects factor and time (baseline, 4-week, and 16-week) as the within-subjects factor. The primary treatment effect was evaluated based on the group × time interaction. Additional within-group changes over time were assessed using repeated-measures ANOVA, while between-group comparisons at individual follow-up time points were performed using independent-samples *t* tests. Statistical significance was set at *p* < 0.05.

## 3. Results

### 3.1. Participant Characteristics

**Figure 1:**
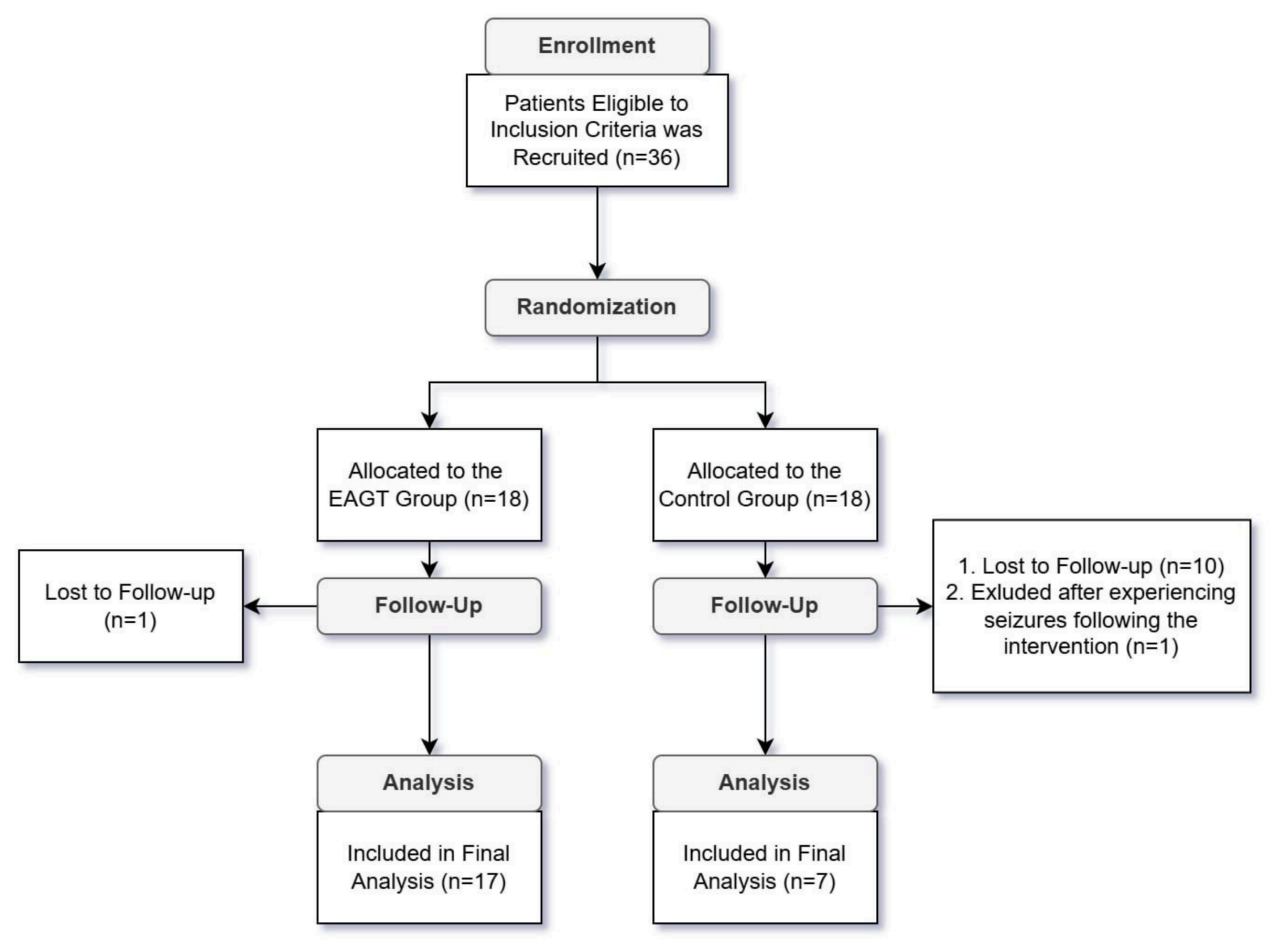
Flowchart of patient recruitment.

Thirty-six patients were enrolled and randomly assigned to the EAGT group (*n* = 18) or the control group (*n* = 18). A preference for the robotic intervention, combined with the inability to blind participants, resulted in higher attrition in the control group. One patient in the EAGT group did not complete follow-up, whereas 10 participants in the control group withdrew; an additional patient in the control group was excluded after experiencing seizures following initiation of the intervention. The final analysis included 17 patients in the EAGT group and 7 in the control group. The flow of participant recruitment was shown in Figure 1.

The baseline characteristics of the participants were presented in Table 1. The two groups did not differ significantly with respect to demographic variables, such as age, sex, stroke etiology, and time since stroke onset, or baseline functional status, as measured by the 10MWT, 6MWT, and FMA-LE.

### 3.2. Therapeutic Effects of EAGT

#### 3.2.1. Primary Outcomes

Table 2 presented the clinical assessments, including the 10MWT, 6MWT, and FMA-LE, conducted at baseline, 4 weeks, and 16 weeks. The two-way mixed ANOVA demonstrated a significant main effect of group for the 6MWT (F = 6.217, *p* = 0.015), whereas no significant group effect was observed for the 10MWT (F = 2.756, *p* = 0.102) or FMA-LE (F = 0.129, *p* = 0.721). A significant main effect of time was observed for the 10MWT (F = 7.774, *p* < 0.001) and 6MWT (F = 5.824, *p* = 0.005), but not for the FMA-LE (F = 2.190, *p* = 0.120). Importantly, no significant group × time interaction was identified for the 10MWT (F = 0.321, *p* = 0.727), 6MWT (F = 0.182, *p* = 0.834), or FMA-LE (F = 0.609, *p* = 0.547), indicating that changes over time did not differ significantly between the EAGT and conventional rehabilitation groups.

Table 3 showed the within-group and between-group comparisons for the clinical outcomes. The EAGT group demonstrated statistically significant within-group improvements from baseline to follow-up across all primary metrics (10MWT: *p* = 0.008; 6MWT: *p* = 0.024; FMA-LE: *p* = 0.023), whereas the control group showed no significant changes across these parameters (10MWT: *p* = 0.052; 6MWT: *p* = 0.113; FMA-LE: *p* = 0.727). Regarding between-group comparisons, no statistically significant differences were observed at either the 4-week follow-up (10MWT: *p* = 0.657; 6MWT: *p* = 0.251; FMA-LE: *p* = 0.567) or the 16-week follow-up (10MWT: *p* = 0.186; 6MWT: *p* = 0.116; FMA-LE: *p* = 0.453).

#### 3.2.2. Secondary Outcomes

Gait analysis results were summarized in Table 4. Longitudinal analysis via ANOVA showed that only gait speed and cadence improved significantly from baseline to the 16-week follow-up (*p* = 0.008 and *p* < 0.001, respectively). Specifically, walking speed increased from 0.3 ± 0.2 m/s at baseline to 0.5 ± 0.2 m/s at 4 weeks and 0.6 ± 0.3 m/s at 16 weeks. Similarly, cadence improved from 19.8 ± 10.3 steps/min to 32.2 ± 12.4 steps/min at 4 weeks and 35.4 ± 12.4 steps/min at 16 weeks. While speed and cadence improved, the intervention did not significantly alter the stride length (*p* = 0.516). Moreover, all measured symmetry indices (step length, stance phase, and swing phase) showed no statistical deviation from baseline (all *p* > 0.05).

## 4. Discussion

Changes in gait speed, walking endurance, and lower-extremity motor function were not significantly different between the EAGT and conventional rehabilitation groups based on the group × time interaction analysis. Although improvements were observed over time, the addition of knee-powered EAGT did not result in significantly greater gains in these functional outcomes. The increase in walking speed after training with a knee-powered overground exoskeleton was accompanied by a higher cadence rather than a longer stride length. However, gait asymmetry was not significantly corrected following such intervention.

To date, several single-arm studies have reported beneficial effects of overground EAGT on gait speed, endurance, lower-extremity strength, and even balance or gait symmetry in patients with subacute stroke [25,26,27]. However, comparative studies with conventional rehabilitation remain limited and have yielded inconsistent results. In a retrospective study evaluating the Keeogo exoskeleton, 17 patients with subacute stroke received EAGT in addition to conventional rehabilitation, whereas 81 matched controls received conventional rehabilitation alone [28]. After six EAGT sessions, both groups demonstrated significant improvements in walking speed and endurance, balance, and activities of daily living. Similar to the present study, overall changes were comparable between groups; however, a greater proportion of patients in the EAGT group achieved the minimal clinically important difference in walking speed (0.16 m per second). In contrast, this pattern was not observed in our cohort, in which 70.6% of participants in the EAGT group and 85.7% in the control group reached this threshold.

In another two randomized controlled trials, an overground exoskeleton with hip and knee actuation was used [29,30]. Improvements in walking speed, walking endurance, and lower-extremity function were comparable between the group receiving EAGT and the group receiving conventional rehabilitation. Yoo et al. [29] found more participants in the EAGT group (5 of 9) recovered limited community ambulation ability than in the conventional rehabilitation group (2 of 8), whereas Molteni et al. [30] found no significant between-group difference in the proportion of independent ambulators. Moreover, a recent meta-analysis of randomized controlled trials comparing overground exoskeletons with traditional therapy found only a small effect size (standardized mean difference: 0.25, 95% CI: −0.04 to 0.53) in gait speed for patients with subacute stroke [31]. Consistent with our findings, current evidence suggests that EAGT produces improvements in walking speed, endurance, and lower-extremity function comparable to those achieved with conventional rehabilitation.

In this study, walking speed and cadence were significantly increased after training, but stride length was not. In a systematic review on post-stroke gait parameter changes, 27 out of 28 studies found an increase in paretic stride length, and 25 out of 30 studies found an increase in cadence after various physical therapy techniques [32]. A meta-analysis by Liu et al. [33], evaluating overground exoskeletons with hip or ankle actuation, showed significant improvements in gait speed, cadence, and step length; notably, hip-powered devices were associated with enhancements in cadence, step length, and temporospatial symmetry. Also, in the case-controlled trial using Keeogo by Huang et al. [28], gait velocity and hip range of motion on the affected side increased after training, implying that kinematic changes may contribute to functional improvement. Collectively, these findings underscore the importance of hip flexion and extension in optimizing walking performance and symmetry. Because Keeogo provides actuation at the knee only, its capacity to adjust step length may be more limited than that of devices incorporating hip assistance.

No significant longitudinal changes in step length, stance phase, or swing phase symmetry were observed in the EAGT group. Interlimb discrepancies are often characterized by a shorter stance time and longer swing time of the affected limb, as well as shorter step length of the unaffected limb, although the opposite can happen [17,34,35]. These temporal–spatial asymmetries are related to the motor function of the affected limb, walking velocity, and standing balance [17,34]. In a cross-sectional study of 194 stroke survivors, the swing time, stance time, and step length asymmetries worsened with chronicity of stroke, whereas gait velocity did not differ significantly across stages [36]. In a longitudinal study of 49 ambulatory stroke survivors, Von Schroeder et al. [37] observed faster gait velocity during the first year after stroke, while temporal asymmetrical gait patterns remained. Likewise, Chow et al. [38] reported significant improvements in gait speed and symmetry within the first six months after stroke, followed by an apparent plateau. Regarding the impacts of overground exoskeletons on gait deviations, Goffredo et al. [39] demonstrated no significant changes in spatial–temporal symmetry after 15 training sessions. Additionally, Huang et al. [28] found no significant between-group difference in joint motion symmetry in patients receiving EAGT as compared with conventional rehabilitation. These findings support the concept that gait asymmetry may represent a compensatory strategy for lower-extremity motor impairment after stroke, and may be resistant to modification, even with EAGT. Once established during the subacute period, patients are unlikely to deviate from preferred gait patterns.

Exoskeleton design is increasingly focused on reducing device size and weight to improve usability [14,40]. Also, enhancing natural movement activation and gait patterns may further deepen human–robot interaction, highlighting the importance of advanced sensors and adaptive control strategies [41]. With the rapidly expanding range of available exoskeletons, the field is shifting from a one-size-fits-all approach toward personalized matching of device characteristics and training parameters to individual users. Notably, integration of artificial intelligence has the potential to substantially boost real-time adaptability to user movement and environmental demands, thereby advancing the precision and effectiveness of exoskeleton-assisted gait training. Keeogo, a device with minimal external constraints and knee-only actuation, requires careful patient selection. In patients with insufficient trunk control or reduced foot clearance, use of such systems could increase fall risk and place greater physical and cognitive burdens on therapists to ensure safety. Further studies examining specific patient subgroups may clarify which robotic systems are most effective for particular clinical profiles.

Several limitations should be considered when interpreting our findings. First, the small sample size likely reduced our statistical power. This may account for the non-significant improvements observed in the control group’s functional tests. Second, substantial differential attrition occurred, with 17 participants completing follow-up in the EAGT group and 7 in the conventional rehabilitation group. During the subacute phase of stroke rehabilitation in Taiwan, transfers between hospitals or rehabilitation facilities are common because of bed availability, proximity to home, and preferences regarding subsequent inpatient rehabilitation. Because treatment allocation could not be blinded, access to the specialized EAGT program may also have provided participants in the intervention group with greater motivation to remain at the study hospital, whereas conventional rehabilitation was more readily available at other facilities. This differential retention may have compromised the balance achieved through randomization, introduced attrition bias, and reduced the precision and generalizability of the estimated treatment effects. Accordingly, the between-group comparisons should be interpreted with caution. Furthermore, accelerometer-based gait analysis was performed only in the EAGT group, precluding direct comparison of temporospatial parameters between groups. Therefore, the observed improvement in cadence should be interpreted as within-group longitudinal changes rather than treatment effects specifically attributable to EAGT. Another limitation was the potential baseline imbalance between groups, particularly given the small sample size. In addition, the relatively large variability in functional outcomes may have masked clinically relevant changes in individual participants. Future studies with larger samples should consider covariate adjustment for important prognostic factors, such as age, sex, baseline gait performance, and time since stroke. Nevertheless, this study yielded valuable insights into gait modifications following robotic training. Finally, as our investigation was exclusively focused on objective gait metrics, activities of daily living (ADL) and patient satisfaction were not assessed. Incorporating these outcomes in future research would provide a more holistic clinical evaluation.

## 5. Conclusions

Preliminary results from this pilot study suggest knee-powered EAGT combined with conventional rehabilitation was associated with improvements in walking performance among patients with subacute stroke, although superiority over conventional rehabilitation was not demonstrated. Within the EAGT group, enhancement in walking speed appeared to be accompanied by increased cadence rather than by changes in stride length or gait symmetry. Larger trials are required to better define the clinical role of knee-powered overground exoskeletons in stroke rehabilitation.

## Figures and Tables

**Table 1:** Comparison of the demographic and baseline functional data between groups.

	EAGT Group (*n* = 17)	Control Group (*n* = 7)	*p*-Value
Age (years)	59.2 ± 9.5	55.3 ± 7.5	0.338
Sex (M/F)	11/6	3/4	0.324
Etiology (*n*)			0.195
Ischemic	14	4	
Hemorrhagic	3	3	
Hemiplegic side (R/L)	9/8	5/2	0.404
Stroke onset (days)	72.1 ± 41.9	62.4 ± 44.0	0.617
10MWT (m/s)	0.3 ± 0.2	0.4 ± 0.3	0.323
6MWT (m)	87.0 ± 60.1	133.1 ± 87.5	0.149
FMA-LE	60.4 ± 9.3	62.9 ± 6.1	0.530

Values are presented as the mean ± SD for continuous variables and counts for categorical variables. EAGT, exoskeleton-assisted gait training; 10MWT, 10-min walk test; 6MWT, 6-m walk test; FMA-LE, Fugl–Meyer Assessment lower-extremity section.

**Table 2:** Functional outcomes and group × time interaction analysis.

Functional Performance	EAGT Group (*n* = 17)			Control Group (*n* = 7)			ANOVA		
	Baseline	4-Week	16-Week	Baseline	4-Week	16-Week	Group	Time	Interaction
10MWT (m/s)	0.3 ± 0.2	0.5 ± 0.2	0.6 ± 0.3	0.4 ± 0.3	0.5 ± 0.3	0.8 ± 0.2	F = 2.756, *p* = 0.102	F = 7.774, *p* < 0.001 *	F = 0.321, *p* = 0.727
6MWT (m)	87.0 ± 60.1	136.3 ± 73.2	161.5 ± 96.2	133.1 ± 87.5	175.3 ± 75.0	229.5 ± 81.5	F = 6.217, *p* = 0.015 *	F = 5.824, *p* = 0.005 *	F = 0.182, *p* = 0.834
FMA-LE	60.4 ± 9.3	67.9 ± 6.5	65.6 ± 7.4	62.9 ± 6.1	66.0 ± 8.9	62.9 ± 9.8	F = 0.129, *p* = 0.721	F = 2.19, *p* = 0.120	F = 0.609, *p* = 0.547

Values are presented as the mean ± SD. EAGT, exoskeleton-assisted gait training; 10MWT, 10-m walk test; 6MWT, 6-min walk test; FMA-LE, Fugl–Meyer Assessment lower-extremity section. * *p* < 0.05; group × time interaction was evaluated using a two-way mixed-design ANOVA.

**Table 3:** Within-group and between-group comparisons of functional outcomes.

	Within-Group Comparison (*p*-Value)		Between-Group Comparison (*p*-Value)	
	**EAGT**	**Control**	**4-Week**	**16-Week**
10MWT (m/s)	0.008 *	0.052	0.657	0.186
6MWT (m)	0.024 *	0.113	0.251	0.116
FMA-LE	0.023 *	0.727	0.567	0.453

EAGT, exoskeleton-assisted gait training; 10MWT, 10-m walk test; 6MWT, 6-minnte walk test; FMA-LE, Fugl–Meyer Assessment lower-extremity section. * *p* < 0.05 by independent repeated-measures ANOVA for the within-group and independent-samples *t* tests for between-group comparisons.

**Table 4:** Gait analysis results of the EAGT group at baseline and follow-up.

	Baseline	4-Week	16-Week	*p*-Value
Speed (m/s)	0.3 ± 0.2	0.5 ± 0.2	0.6 ± 0.3	0.008 *
Cadence (steps/min)	19.8 ± 10.3	32.2 ± 12.4	35.4 ± 12.4	<0.001 *
Stride length (m)	1.2 ± 0.3	1.3 ± 0.3	1.4 ± 0.4	0.516
Step length SI	0.2 ± 0.2	0.1 ± 0.1	0.1 ± 0.1	0.297
Stance phase SI	0.2 ± 0.2	0.2 ± 0.2	0.2 ± 0.1	0.963
Swing phase SI	0.4 ± 0.3	0.3 ± 0.3	0.4 ± 0.2	0.952

Values are presented as the mean ± SD. SI, symmetry index. * *p* < 0.05 by repeated-measures ANOVA comparisons.

## Data Availability

The datasets used and/or analyzed during the current study are available from the corresponding author on reasonable request.
